# Hippo/YAP signaling pathway in colorectal cancer: regulatory mechanisms and potential drug exploration

**DOI:** 10.3389/fonc.2025.1545952

**Published:** 2025-06-19

**Authors:** Lingqiu Zhang, Fan Zhang, Haimei Liang, Xiangling Qin, Yuemi Mo, Shiyi Chen, Jinling Xie, Xiaotao Hou, Jiagang Deng, Erwei Hao, Zhengcai Du

**Affiliations:** ^1^ Guangxi Key Laboratory of Efficacy Study on Chinese Materia Medica, Guangxi University of Chinese Medicine, Nanning, China; ^2^ University Engineering Research Center of Reutilization of Traditional Chinese Medicine Resources, Guangxi University of Chinese Medicine, Nanning, China; ^3^ Guangxi Key Laboratory of TCM Formulas Theory and Transformation for Damp Diseases, Guangxi University of Chinese Medicine, Nanning, China; ^4^ Institute of Traditional Chinese and Zhuang-Yao Ethnic Medicine, Guangxi University of Chinese Medicine, Nanning, China; ^5^ Guangxi International Zhang Medicine Hospital Affiliated to Guangxi University of Chinese Medicine, Nanning, China

**Keywords:** colorectal cancer, Hippo pathway, YAP, targeted therapy, tumor microenvironment

## Abstract

**Objective:**

Colorectal cancer (CRC) remains a leading cause of cancer-related morbidity and mortality worldwide. The Hippo signaling pathway, particularly its downstream effector Yes-associated protein (YAP), has been identified as a pivotal regulator of CRC tumorigenesis, metastasis, drug resistance, and tumor microenvironment remodeling. This review aims to comprehensively synthesize recent advances in the regulatory mechanisms of the Hippo/YAP pathway and critically evaluate its therapeutic implications, including emerging clinical interventions and epigenetic modulation.

**Methods:**

A systematic literature review was conducted to synthesize mechanistic studies, translational research, and clinical trials involving the Hippo/YAP pathway in CRC. We focused on elucidating its upstream and downstream interactions, crosstalk with other signaling cascades, and the dual oncogenic/tumor-suppressive roles of YAP/TAZ. Epigenetic regulatory mechanisms (e.g., DNA methylation, histone modifications) and non-coding RNA-mediated regulation were rigorously analyzed. Additionally, therapeutic strategies targeting the Hippo pathway—including clinical agents, molecular inhibitors, non-coding RNAs (ncRNAs), and natural products—were systematically evaluated to assess their clinical potential.

**Results:**

Hippo pathway dysregulation drives CRC progression through aberrant YAP activation, which promotes tumor proliferation, metastasis, metabolic reprogramming, and immune evasion. Notably, emerging evidence reveals context-dependent tumor-suppressive functions of YAP/TAZ in specific CRC subtypes, such as via suppression of Wnt signaling. Epigenetic mechanisms, including DNA methylation and histone modifications, further fine-tune YAP activity. Preclinical and clinical investigations highlight the efficacy of diverse Hippo/YAP-targeted interventions, with recent clinical trials (e.g., VT3989, IK-930, IAG933, ION537) underscoring the translational promise of this pathway.

**Conclusions:**

The Hippo/YAP axis serves as a central hub in CRC biology, exhibiting context-dependent dual roles in both oncogenesis and tumor suppression. Integrating cutting-edge insights into its regulatory networks and clinical targeting offers novel perspectives for precision oncology. By bridging fundamental discoveries with translational applications, this review establishes Hippo/YAP as a compelling therapeutic target and provides a theoretical foundation for developing innovative CRC therapies.

## Introduction

Colorectal cancer (CRC) is one of the most common malignant tumors worldwide, with both incidence and mortality rates ranking among the highest. According to the latest Global Cancer Statistics Report (2024), approximately 20 million new cancer cases were diagnosed globally in 2022. Among these, CRC accounted for 1.93 million new cases and 904,000 deaths, representing 9.6% of global cancer incidences and 9.3% of cancer-related deaths, respectively (1). The incidence and mortality rates vary across regions and countries, potentially due to differences in genetic predisposition, dietary habits, and lifestyle-related risk factors (2–5). CRC is characterized by challenges in early diagnosis, high metastasis rates, and frequent recurrence, contributing to its poor prognosis. Therefore, elucidating the pathogenesis and influencing factors of CRC, and exploring new therapeutic targets are the challenges and hotspots in CRC research.

Current CRC treatment strategies include surgery, chemotherapy, radiotherapy, targeted therapy, and immunotherapy. However, these approaches face significant limitations, such as treatment resistance and adverse side effects (6, 7). Advances in precision medicine, genomics, and proteomics have opened new possibilities for early diagnosis and personalized therapy for CRC.

Recent studies have unveiled the “dual-module” signal transduction model of the Hippo pathway (HPO1 and HPO2), which cooperatively regulates LATS1/2 and YAP/TAZ activities, influencing cell fate, organ size, and tumor progression (8, 9).

Dysregulation of the Hippo pathway has been strongly associated with the development and progression of various cancers, including lung, liver, breast, and nasopharyngeal carcinomas (10–13), as well as CRC (14). Notably, Hippo/YAP signaling exhibits elevated activity in CRC compared to normal tissues (15). Increased YAP protein expression has been linked to liver metastasis and tumor recurrence in CRC patients (16, 17). Furthermore, PLA2G16 has been shown to decrease p-YAP and p-TAZ protein expression, disrupting Hippo pathway balance and promoting CRC progression (18). These findings highlight the Hippo signaling pathway as a critical regulatory network in CRC, providing a promising therapeutic strategy for its treatment.

This review systematically explores the roles and regulatory mechanisms of the Hippo/YAP signaling pathway in CRC. It summarizes the latest research progress on clinical drugs, molecular inhibitors, non-coding RNAs (ncRNAs), and natural products that target this pathway for CRC inhibition. Emerging strategies for YAP-targeted drug development demonstrate significant therapeutic potential, offering new directions for precision medicine and personalized treatment of CRC. By targeting critical nodes of the Hippo signaling pathway, these strategies lay the foundation for tailored therapeutic approaches that cater to the unique needs of CRC patients.

## Hippo signaling pathway

The Hippo signaling pathway, also known as the Salvador/Warts/Hippo (SWH) pathway, was initially discovered in Drosophila melanogaster and named after its core component, the Hippo protein kinase (19, 20). This signaling pathway demonstrates a high degree of evolutionary conservation from fruit flies to mammals, reflecting its significant function within biological systems (21). Its core components include upstream kinases such as mammalian STE20-like protein kinases (MST1/2) and their adaptor protein Salvador (SAV1), intermediate kinases large tumor suppressor kinases 1/2 (LATS1/2) with their scaffold proteins MOBKL1A/B (MOB1A/B), and downstream effectors Yes-associated protein (YAP) and transcriptional coactivator with PDZ-binding motif (TAZ), along with the TEAD transcription factors (22, 23). These components work synergistically to regulate cellular behavior through a signaling cascade, ensuring tissue homeostasis and preventing abnormal cell proliferation.

**Figure 1:**
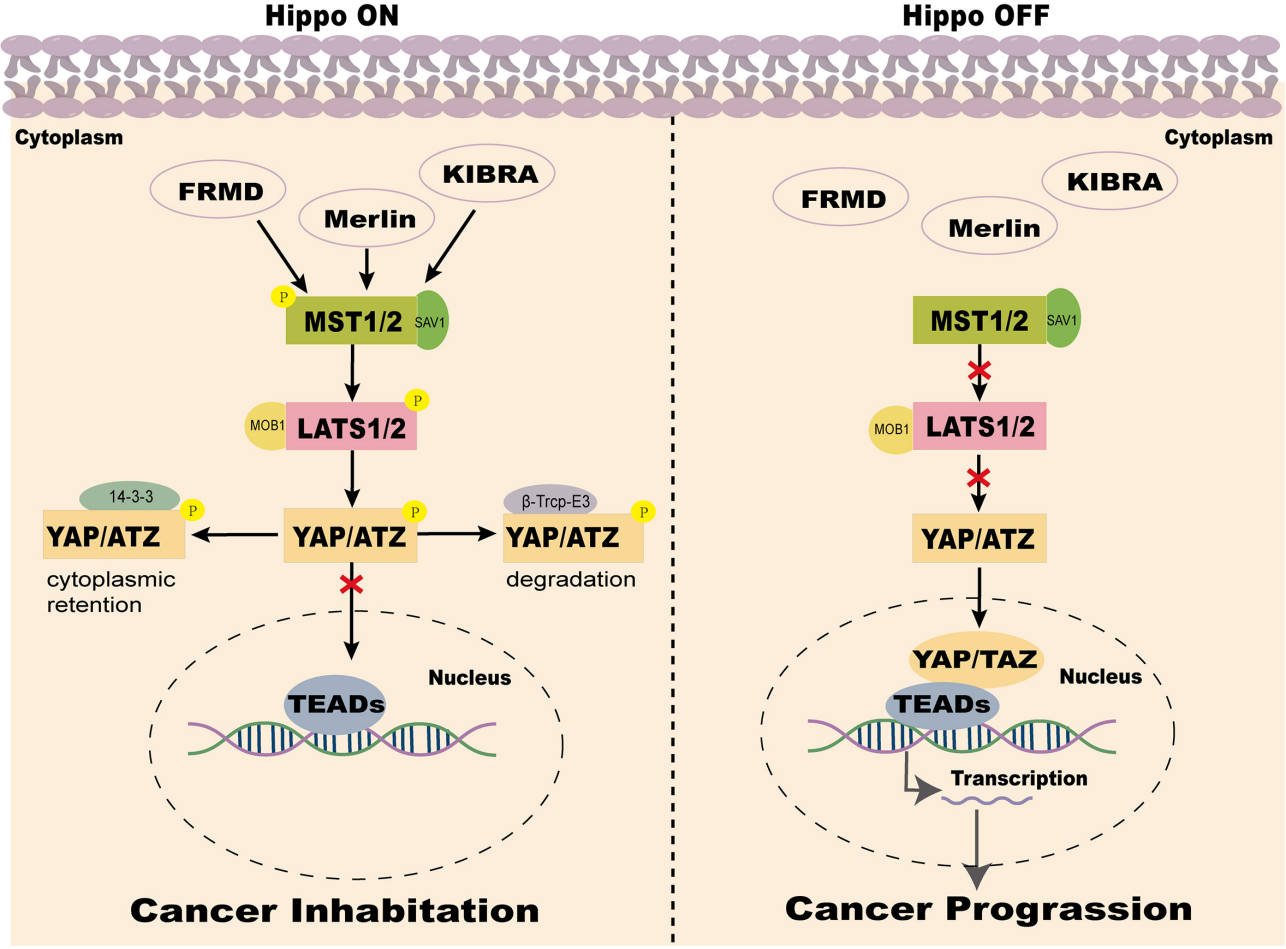
Two states of the Hippo pathway in the mammalian tumor microenvironment. Note: When the Hippo signaling pathway is activated, MST1/2 kinases, together with SAV1, phosphorylate and activate LATS1/2. LATS1/2, in conjunction with MOB1A/B, phosphorylates YAP and TAZ. The phosphorylated YAP/TAZ binds to 14-3–3 proteins and is sequestered in the cytoplasm, leading to suppression of YAP/TAZ activity. Additionally, phosphorylated YAP/TAZ undergoes ubiquitination mediated by casein kinase 1 (CK1) and β-transducin repeat-containing protein (β-TrCP), followed by proteasomal degradation. Conversely, when the Hippo signaling pathway is inactivated or suppressed, YAP/TAZ becomes dephosphorylated and activated, translocates into the nucleus, and binds to TEAD transcription factors. This interaction drives the transcription of oncogenic genes, promoting tumor growth, proliferation, and migration.

The Hippo pathway can be activated by various intracellular and extracellular signals, including cell-cell contact, extracellular matrix (ECM) signals, cell density, mechanical forces, cellular energy status, and stress signals. Additionally, metabolic changes, mitogenic signals, tyrosine kinase receptor signaling, and G protein-coupled receptor (GPCR) ligands significantly influence its activity (24, 25). During signal transduction, MST1/2 kinases, assisted by SAV1, activate LATS1/2 kinases, which phosphorylate YAP/TAZ to regulate their subcellular localization and subsequently activate the Hippo pathway (26, 27). When the Hippo pathway is inhibited, unphosphorylated YAP/TAZ translocates into the nucleus, where they bind with TEAD transcription factors to regulate the expression of target genes associated with cell growth, proliferation, and apoptosis (28–30) (**Figure 1**).

YAP is a key transcriptional coactivator in the Hippo signaling pathway, responsible for initiating the transcription of downstream target genes. The YAP protein is encoded by a gene located on human chromosome 11q22 and exists in two splice isoforms: YAP1 and YAP2. YAP1 contains a single WW domain, whereas YAP2 contains two WW domains. These WW domains are critical for binding to phosphorylated proteins and play a crucial role in signal transduction and protein-protein interactions. Rich in proline residues, YAP interacts with various proteins and participates in multiple cellular signaling pathways, thereby executing diverse biological functions such as cell proliferation, differentiation, and apoptosis (27, 31, 32). Since YAP lacks conventional DNA binding domains, its function as a transcriptional co-activator relies on interactions with transcription factors such as TEAD to regulate the expression of target genes. Within the Hippo signaling pathway, YAP activity is tightly regulated by upstream kinases, including MST1/2 and LATS1/2. Upon activation of these kinases, YAP is phosphorylated and rendered inactive, sequestered in the cytoplasm, thereby inhibiting its transcriptional activity and ultimately restricting cell growth and proliferation (33). Studies have shown that LOXL1 suppresses YAP’s nuclear transcriptional function in CRC by inducing MST kinase activity, thereby inhibiting the malignant progression of the cells (34). These findings highlight the central role of YAP in the Hippo signaling pathway and highlight its critical importance in regulating cell proliferation and tumor development.

## Hippo/YAP signaling pathway in CRC

**Figure 2:**
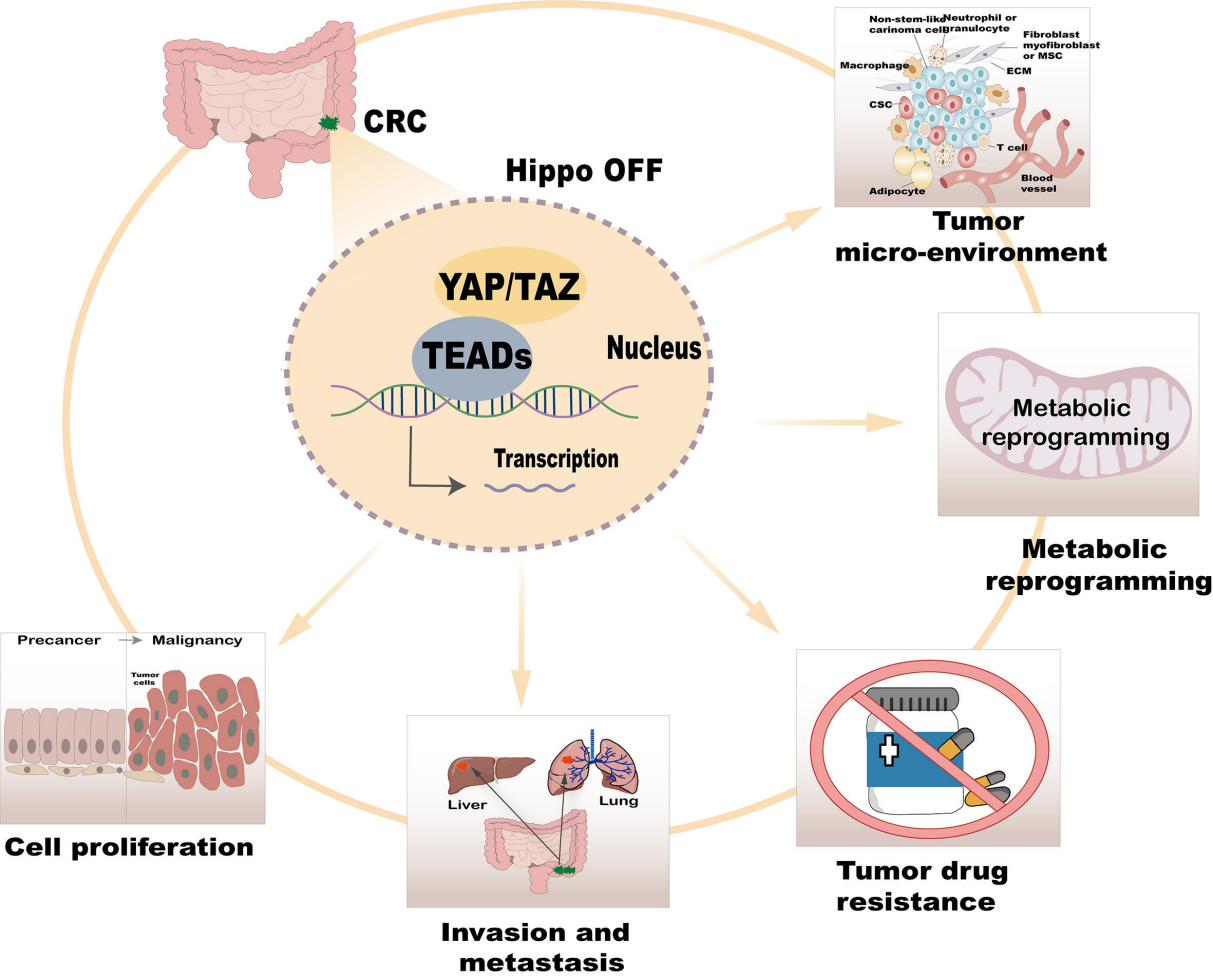
Regulatory role of the Hippo/YAP pathway in CRC.

The Hippo/YAP signaling pathway serves as a critical cellular regulatory network, playing multifaceted roles in CRC. These include promoting cell proliferation, invasion, metastasis, drug resistance, metabolic reprogramming, and regulation of the tumor microenvironment (**Figure 2**). Recent advancements in the study of this pathway have not only elucidated its underlying mechanisms in tumor biology but also identified potential drug targets and novel strategies for CRC treatment. Below, we systematically explore the regulatory functions of the Hippo/YAP pathway in CRC.

### Promotion of CRC cell proliferation

Cell proliferation is fundamental to the growth, development, and maintenance of tissue homeostasis in organisms. Aberrant proliferation is a hallmark of cancer. Studies have revealed that YAP is highly expressed in the nucleus of CRC cells (15).

and plays a pivotal role in promoting CRC initiation and progression (35, 36). Through its transcriptional activation function, YAP drives tumor progression (37), YAP enhances the expression of genes such as AJUBA, WTIP, and SAMD4A by binding to TEAD transcription factors, which promotes the formation and abundance of P-bodies. Knocking out YAP significantly reduces the number of P-cells, thereby impairing cancer cell proliferation and migration. Another study revealed that hypoxia-inducible factor-2α (HIF-2α) markedly increases YAP expression and activity in CRC cell lines and mouse models, thereby enhancing the transcriptional activity of downstream genes and promoting tumorigenesis (38). Conversely, ARRDC3 suppresses CRC cell proliferation by facilitating YAP degradation (39). These findings highlight the regulatory role of YAP in CRC proliferation and highlight its potential as a therapeutic target. Targeting YAP offers promising avenues for the development of novel CRC treatment strategies.

### Promotion of CRC invasion and metastasis

Epithelial-mesenchymal transition (EMT) is a crucial initial step for tumor cell invasion and metastasis. During EMT, epithelial cells lose their polarity and cell-cell adhesion properties, while acquiring mobility and invasiveness. This transition enables tumor cells to infiltrate surrounding tissues, enter the vasculature, and extravasate at distant sites to form secondary metastatic foci (40). Studies have shown that the YAP-TEAD1 complex positively regulates the EMT process in circulating tumor cells (CTCs), promoting liver metastasis in CRC (41). YAP enhances the transcriptional activity of genes such as SDC2, HACE1, N-cadherin, and Vimentin, thereby driving the EMT process and significantly increasing CRC cell migration and invasion capabilities (17, 42, 43).

In conclusion, YAP plays a pivotal role in transitioning CRC cells from the micrometastatic stage to the active growth stage (44–46). Additional research has shown that silencing YAP suppresses CRC cell invasion (47), the complete loss of YAP correlates with poor prognosis in CRC (48). These findings highlight the multifaceted regulatory function of YAP and related molecules in CRC metastasis and invasion.

### Regulation of drug resistance in tumors

Current treatments for CRC include surgical resection and chemotherapy. However, the development of resistance to chemotherapeutic agents during treatment frequently leads to disease progression. Overexpression of YAP protein in CRC has been closely linked not only to tumor initiation and progression but also to resistance to chemotherapeutic drugs (49, 50). It has been shown that the YAP inhibitor Verteporfin enhances the sensitivity of 5-FU resistant cells to 5-FU by disrupting the YAP-TEAD complex (51). Additionally, YAP positively regulates the expression of cyclooxygenase-2 (COX-2), thereby increasing CRC cell resistance to paclitaxel. To counteract this, researchers have developed a dual inhibitor, GCCSysm-4 (G-4), which simultaneously inhibits YAP and COX-2 activity, significantly enhancing the sensitivity of CRC cells to chemotherapeutic agents (52). Reducing YAP expression has also been found to improve CRC cell sensitivity to cetuximab. Simvastatin enhances the cytotoxicity of EGFR inhibitors against CRC cells by inhibiting YAP nuclear translocation and reducing its total protein level. Furthermore, the combination of simvastatin with EGFR inhibitors has proven more effective than monotherapy (53). These findings highlight YAP as a key regulatory molecule in drug resistance and suggest potential strategies for overcoming chemotherapeutic resistance.

### Regulation of metabolic reprogramming

Metabolic reprogramming is a hallmark of cancer and an important potential target for tumor therapy. To sustain rapid growth and metastasis, cancer cells undergo significant metabolic reprogramming. Studies have demonstrated that YAP significantly influences CRC aggressiveness and stemness through the regulation of various metabolic pathways. For instance, mutations in the KRAS gene, a common oncogenic driver, affect YAP function via specific molecular mechanisms. These mutations increase YAP stability in the cytoplasm, enabling its transcriptional activation of glycolytic genes, including GLUT3 (glucose transporter 3), which enhances the invasiveness and stemness of CRC cells (54). Additionally, YAP promotes cholesterol biosynthesis by regulating the expression of ZMYND8 to activate the mevalonate (MVA) pathway (55). Furthermore, microRNA miR-103a-3p induces aerobic glycolysis by targeting the YAP/HIF1α pathway, providing CRC cells with additional metabolic support for proliferation (56).

These findings suggest that targeting YAP and its associated metabolic pathways represents a promising therapeutic strategy for CRC treatment, addressing the metabolic vulnerabilities inherent in cancer progression.

### Regulation of the tumor microenvironment

The tumor microenvironment (TME) provides physical and metabolic support to tumor cells, facilitating their metastasis to distant organs (57). Within the TME, the programmed death receptor 1 (PD-1) and its ligand, programmed death-ligand 1 (PD-L1), play critical roles. These molecules are key mediators of tumor immune evasion and serve as important biomarkers for immunotherapy. Tumor cells can evade immune surveillance by overexpressing PD-L1, which suppresses T-cell activation. PD-1/PD-L1 immune checkpoint inhibitors have been clinically approved for the treatment of several malignancies, including non-small cell lung cancer and malignant melanoma, demonstrating their efficacy and feasibility (58–60). In CRC, YAP directly binds to TEAD family proteins to activate the promoter of the PD-L1 gene, thereby upgrading PD-L1 expression and enhancing immune evasion (61). Reducing YAP expression or activity diminishes the transcriptional activity of the PD-L1 promoter, thereby impairing the immune evasion capacity of CRC cells (62). These findings highlight YAP as a potential therapeutic target for CRC immunotherapy.

## The core effector YAP regulates colorectal cancer

### Direct regulation of YAP

As the central effector molecule of the Hippo pathway, YAP’s expression level, subcellular localization, and transcriptional coactivator activity critically regulate the transcription of genes involved in cell proliferation, apoptosis, and epithelial-mesenchymal transition (EMT). These processes are closely linked to tumorigenesis and cancer progression. Fine-tuned regulation of the Hippo signaling pathway—particularly through transcription, translation, post-translational modifications, subcellular localization, and transcriptional coactivation of YAP-is essential for the development of targeted therapies for CRC.

Transcriptional Regulation, a key step in gene expression, occurs at the DNA level and controls mRNA synthesis through interactions between transcription factors and specific DNA sequences, such as promoters and enhancers, either enhancing or repressing transcription (63). Translational regulation operates at the mRNA level and controls protein synthesis. For instance, the 5′ untranslated region (5′ UTR) or 3′ untranslated region (3′ UTR) of certain mRNAs can bind specific proteins, affecting translation efficiency. Studies have shown that miR-375-3p binds to the 3′ UTR of YAP1 mRNA, inhibiting its translation and thereby suppressing CRC progression (64). Post-Translational Modifications (PTMs) chemically modify proteins after synthesis, altering their activity, stability, subcellular localization, and protein-protein interactions. Studies of YAP PTMs have focused on phosphorylation and ubiquitination. Proteins such as DUSP10, SNHG29, and VASN inhibit YAP phosphorylation, promoting its nuclear translocation and driving CRC initiation and metastasis (65–67). Conversely, FA2H and SCRIB enhance YAP phosphorylation, leading to its degradation and reduced transcriptional activity, thereby suppressing CRC cell proliferation (68, 69). Molecules such as Siah1, USP47, and USP7 promote YAP deubiquitination, enhancing its stability and fostering CRC progression (70–72). These three regulatory layers are interconnected and collectively determine the expression patterns and functional states of proteins within the cell, thus fine-tuning cellular behavior and physiological function. Activation beyond gene expression, certain proteins directly influence YAP nuclear translocation and activation of downstream target genes, SNHG14 and KRAS enhance YAP nuclear localization, increasing the transcription of oncogenes and accelerating CRC progression (73, 74). Proteins such as HHEX, MYL9, ANKHD1, and MALAT1 enhance YAP’s transcriptional activity, further promoting the expression of downstream target genes and driving CRC development (75–77). PTPRU and miR-550a-3-5p suppress YAP’s transcriptional activity, thereby limiting downstream oncogene expression and hindering CRC progression (78, 79).

In general, the functional regulation of YAP involves multiple levels of mechanisms such as transcription, post-translational modifications, and subcellular localization, which interact with each other to jointly regulate the activity of YAP and promote the occurrence and development of CRC. A summary and classification of the molecules that directly regulate YAP in CRC, as currently discovered or under investigation, are shown in **Table 1**.

**Table 1:** Molecules directly targeting YAP and their functional roles in regulating CRC.

Regulation Level	Molecule Name	Function	Reference
Transcription	TRIM24	Binds to the promoter of YAP and activates its transcription	(63)
Translation	miR-375-3p	Binds to the 3'UTR of YAP1 mRNA, inhibiting its translation	(64)
	DUSP10	Binds to the Ser397 site of YAP1, promoting YAP dephosphorylation	(65);
Post-translational Phosphorylation Modification	SNHG29	nhibits phosphorylation of YAP at the Ser127 site, promoting upregulation of PD-L1 expression	(66, 67)
	FA2H	Promotes phosphorylation of YAP1 at the Ser127 site, inhibiting its transcriptional activity and slowing CRC cell proliferation	(68)
	VASN	Interacts with YAP protein, inhibiting its phosphorylation and driving CRC initiation and migration	(66)
	SCRIB	Promotes YAP phosphorylation, suppressing CRC invasion and metastasis	(69)
Post-translational Ubiquitination Modification	USP47, USP7	Promotes deubiquitination of YAP, driving CRC progression	(71, 72)
Cellular Subcellular Localization	SNHG14	Increases nuclear localization of YAP, regulating transcription of the proto-oncogene KRAS and promoting CRC proliferation	(73)
	KRAS	Increases the nuclear abundance of YAP, upregulating specific amino acid transporter expression and driving CRC cell metabolism and proliferation	(74)
Transcriptional Co-activation Effect	HHEX	Directly interacts with the YAP-TEAD complex to enhance transcriptional activity	(75)
	MYL9, ANKHD1, MALAT1	Enhance YAP transcriptional activity	(76, 77)
	PTPRU	Inhibits YAP transcriptional activity	(78)
	miR-550a-3-5p	Inhibits YAP/TAZ transcriptional activity, increasing CRC sensitivity to the BRAF inhibitor vemurafenib	(79)

### Indirect regulation of YAP

**Figure 3:**
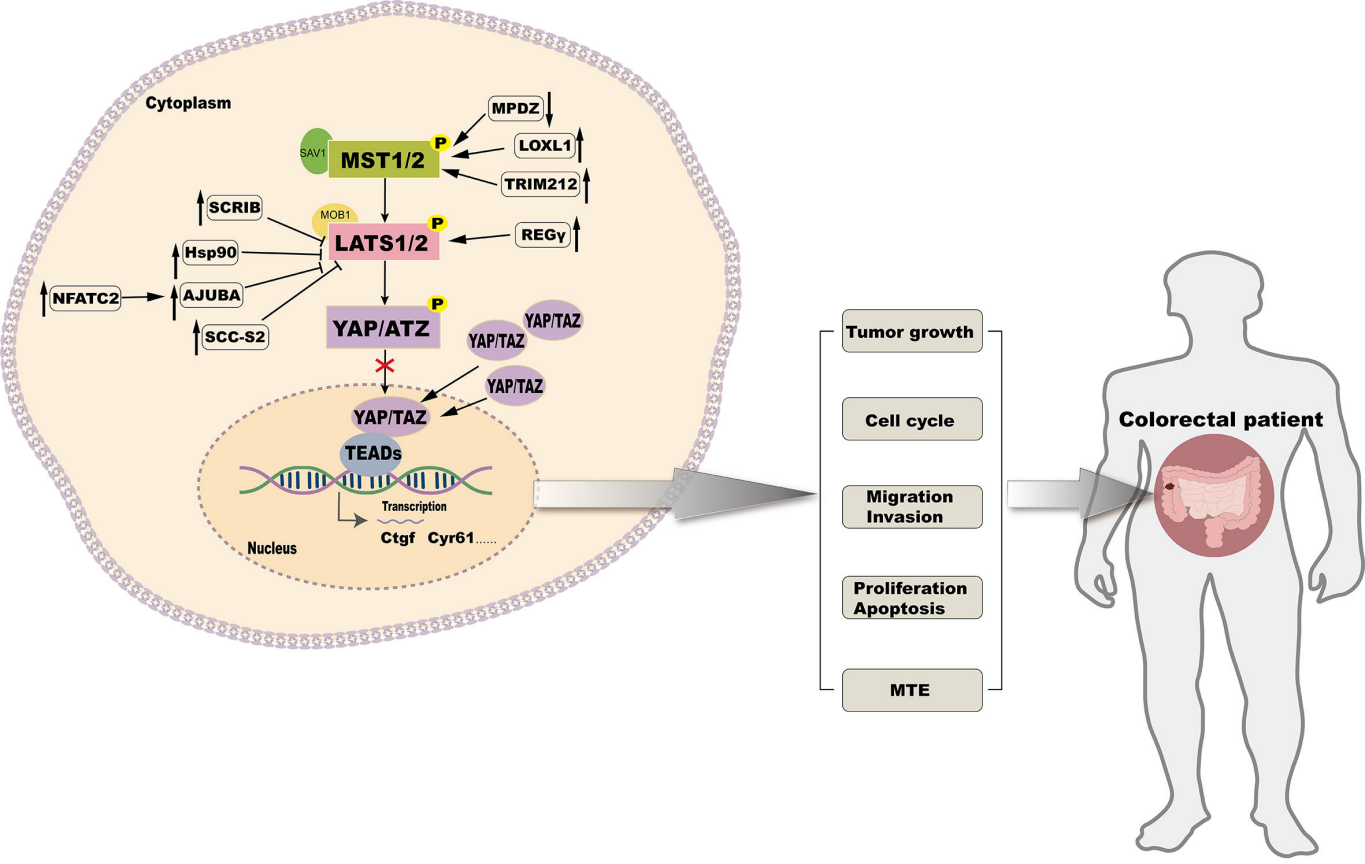
Targeting upstream kinases of the Hippo signaling pathway to regulate CRC.

Subcellular localization and expression of YAP, a key transcriptional coactivator, is tightly regulated by upstream kinases MST1/2 and LATS. By targeting the upstream kinases in the Hippo signaling pathway, YAP activity can be indirectly modulated to achieve anti-CRC effects (**Figure 3**).

SCRIB modulates the Hippo signaling pathway by downregulating the expression of LATS1/2 and MOB1A/B, which leads to a reduction in YAP phosphorylation. This reduction in phosphorylation facilitates the translocation of YAP into the cell nucleus, where it activates oncogenic transcription factors, thereby promoting the proliferation and metastasis of CRC cells (68). Overexpression of Hif-1α under hypoxic conditions enables its direct interaction with the Hsp90 promoter, leading to elevated Hsp90 expression. Hsp90 binds directly to LATS1, resulting in YAP dephosphorylation and nuclear translocation, promoting CRC cell proliferation (80). High levels of NFATC2 increase the expression of AJUBA, which inhibits the activity of the LATS1/2 kinase. This reduces YAP phosphorylation, enhances CRC stemness, and promotes tumor progression (81). In CRC, the overexpression of LOXL1 activates MST1/2 kinases, which promotes the phosphorylation of YAP at multiple sites. This leads to the sequestration of YAP in the cytoplasm or its degradation through the ubiquitination pathway, thereby reducing its nuclear activity and inhibiting the proliferation, migration, and invasion of CRC cells (34). TRIM21 interacts with MST2, enhancing its kinase activity, which retains and degrades YAP in the cytoplasm, thereby suppressing its transcriptional activity and target gene expression, effectively inhibiting CRC invasion and metastasis (82). The knockdown of MPDZ increases LATS1 expression and enhances YAP phosphorylation at Ser127. This reduces its nuclear accumulation, inhibiting YAP transcriptional activity and suppressing CRC cell growth (62). Another study has found that SCC-S2 promotes the proliferation and invasion of CRC cells by inhibiting the kinase activity of LATS1, leading to the dephosphorylation and accumulation of YAP, which in turn activates oncogenes within the nucleus (83). Interaction between REGγ and LATS1 accelerates LATS1 degradation, activating YAP and promoting CRC proliferation (84). The overexpression of Claudin-2 leads to a decrease in the expression of miR-222-3p, which releases the inhibition on YAP target genes, thereby increasing the processes of self-renewal, proliferation, and invasion in CRC cells (85). The loss of ABHD5 facilitates the nuclear translocation of DPY30, activates the SET1A complex, and enhances YAP methylation. This modification maintains CRC stemness capacity (86).

Therapeutic implications these findings reveal that YAP activity is regulated by multiple factors that affect its phosphorylation status, subcellular localization, and transcriptional activity, ultimately affecting CRC progression and metastasis. Targeting these regulatory molecules or restoring their function could provide novel strategies for CRC therapy. By targeting the upstream regulators of YAP, therapeutic interventions may indirectly modulate YAP activity, thereby opening avenues for innovative and effective therapies.

## CRC crosstalk between Hippo/YAP and other signaling pathways in regulating CRC

**Figure 4:**
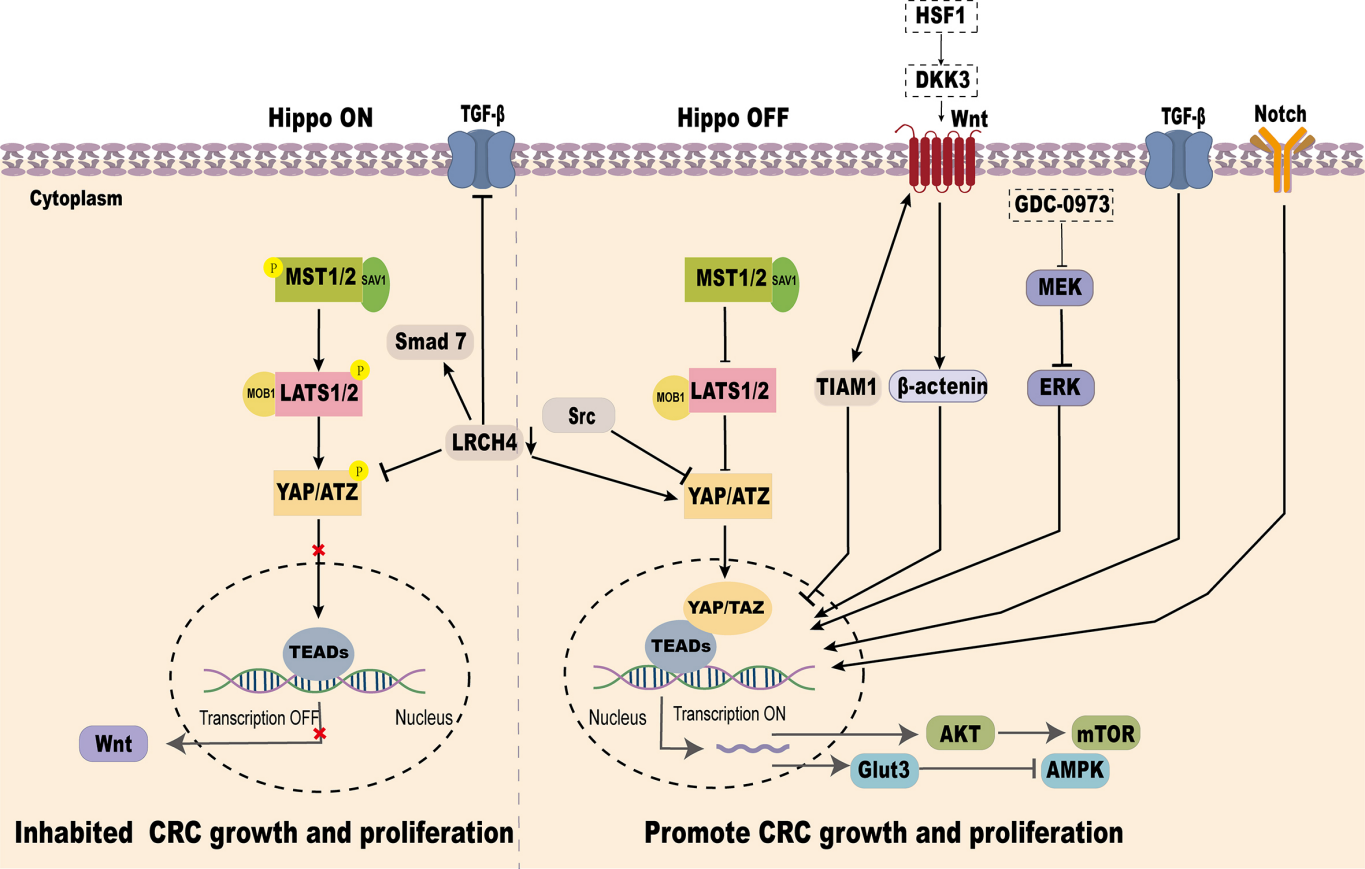
Crosstalk between the Hippo signaling pathway and other pathways in regulating YAP.

YAP, as a key effector of the Hippo signaling pathway, plays a pivotal role in CRC progression. It has been demonstrated that Hippo/YAP interacts with several signaling pathways, including Wnt/β-catenin, TGF-β/Smad, Glut3/AMPK, Akt-mTOR, Notch, and MEK/ERK, to regulate CRC development and metastasis (**Figure 4**).

### YAP and Wnt/β-catenin signaling pathway

Abnormal activation of the Wnt signaling pathway is a common molecular event in CRC, with over 90% of CRC cases exhibiting mutations activating this pathway (87).

YAP-TEAD transcription factor acts as a key downstream effector of Wnt signaling in the gut. Wnt signaling transcriptionally induces the expression of YAP and TEAD1/2/4, promoting intestinal stem cell proliferation and differentiation in a Src-family kinase-dependent manner (88). In the nucleus, TIAM1 inhibits the interaction between TAZ/YAP and TEAD, reducing EMT-related gene expression (AMOTL2, ANKRD1, AXL, CTGF, and CYR61), Wnt pathway activation facilitates TIAM1 nuclear translocation, thereby reducing CRC invasion and migration (89). Conversely, in KRAS-mutant CRC cell lines, MEK inhibitors block MEK-ERK signaling, enhancing YAP nuclear accumulation and activating WNT target genes such as AXIN2, highlighting the intricate crosstalk between YAP and Wnt signaling in CRC progression (90).

### YAP and TGF-β/Smad signaling pathway

TGF-β is a multifunctional cytokine acting as a tumor suppressor in early stages of cancer but promoting tumor progression through EMT in later stages (91). The interplay between TGF-β/Smad signaling and YAP influences CRC progression. Knockdown of LRCH4 suppresses YAP activity, reducing its enhancement of TGF-β/Smad signaling, thereby slowing EMT and CRC invasion (92). Moreover, certain genetic variations in CRC activate YAP/TAZ through TGF-β signaling, triggering transcriptional reprogramming and lineage reversal. This results in tumor cells acquiring independence from the Wnt pathway and resistance to Wnt inhibitors (93).

### YAP and Glut3/AMPK signaling pathways

Glut3, a glucose transporter, facilitates glucose uptake for energy and biosynthesis. In CRC, GLUT3 is highly expressed and associated with poor clinical outcomes (54, 94). AMPK, a key energy sensor, is often suppressed in CRC, promoting tumor growth by altering metabolism and epigenetics (95). YAP overexpression in CRC enhances Glut3-mediated glucose uptake and ATP production, potentially lowering AMPK activity, thus promoting CRC proliferation and migration (96).

### YAP and Akt-mTOR signaling pathway

As an upstream effector molecule, SAV1 can inhibit the Akt-mTOR signaling pathway by inhibiting the activity of YAP, thus preventing the occurrence of CRC (97). Conversely, VASN interacts with YAP to activate the YAP/TAZ pathway, inhibit PTEN, and enhance Akt activity, promoting CRC development (66). Additionally, ANKHD1 or MALAT1 can interact with YAP, thereby activating AKT, increasing the expression and activity of DNA damage repair proteins, promoting DNA repair, reducing radiation-induced cell death, and enhancing the tolerance of tumor cells to radiotherapy (77).

### YAP and notch signaling pathway

In tumor cells, overactivation of Notch signaling and high YAP expression often co-occur, driving cell proliferation and survival. In intestinal epithelial cells, Notch activation combined with p53 loss induces a regenerative cell state characterized by elevated YAP and MLL1 expression, maintaining proliferation and self-renewal, which contribute to CRC progression (98).

### YAP and MEK/ERK signaling pathway

The interplay between YAP and the MEK/ERK pathway is critical for PD-L1 expression in CRC. Activation of the MEK/ERK pathway enhances YAP transcriptional activity, directly upgrading PD-L1 expression. This interplay highlights the intricate signaling network within the tumor microenvironment of CRC (99). The extensive interplay between the Hippo/YAP pathway and other signaling cascades underscores its multifaceted regulatory functions in CRC development and progression. Deciphering these interactions offers critical insights into CRC pathogenesis and unveils opportunities for combinatorial therapeutic strategies targeting these interconnected pathways.

## Epigenetic regulation of the Hippo/YAP pathway in CRC

Epigenetic modifications represent a critical layer of regulation for the Hippo/YAP signaling pathway in CRC, influencing YAP/TAZ expression, localization, and activity. Among these, DNA methylation of upstream tumor suppressors within the Hippo pathway is frequently observed in CRC. For instance, promoter hypermethylation of RASSF2 has been reported in approximately 86% of colon cancer cases, leading to its transcriptional silencing and subsequent YAP/TAZ activation (100). Other key components, including RASSF1, MST1/2, and LATS1/2, are also subject to methylation-dependent inactivation, thereby contributing to the loss of Hippo signaling function.

Histone modifications further fine-tune YAP activity. The histone methyltransferase SETD1A (SET1A) mono-methylates YAP at lysine-342, a modification that prevents its nuclear export and enhances its transcriptional activity (101). In contrast, SIRT1, a histone deacetylase, deacetylates YAP (e.g., at Lys-494), facilitating its cytoplasmic retention and functional inhibition (102). These post-translational histone modifications demonstrate a direct link between the chromatin landscape and Hippo pathway output.

Non-coding RNAs (ncRNAs), including long non-coding RNAs (lncRNAs), microRNAs (miRNAs), and circular RNAs (circRNAs), add an additional layer of epigenetic regulation. miRNAs directly or indirectly target core Hippo components to modulate YAP activity. For instance, miR-429 and miR-103a-3p downregulate LATS2, reducing YAP/TAZ phosphor rylation and promoting their nuclear accumulation and transcriptional activity (56, 103). Conversely, miR-375-3p, which is often downregulated in CRC, directly targets YAP1 mRNA, and its restoration suppresses YAP expression and enhances chemosensitivity (64).Other miRNAs, such as miR-195, miR-22-3p, and miR-372-3p, modulate CRC progression by targeting YAP or its upstream regulators like SAV1 and LATS2 (104–106). Additionally, the downregulation of miR-874-3p is associated with chemotherapy resistance in CRC (107).

LncRNAs and circRNAs, such as AGAP2-AS1, suppress LATS1 phosphorylation, thereby activating YAP (108). Similarly, CAF-sEVsWEE2-AS1 promotes MOB1A degradation, enhancing YAP nuclear localization and transcriptional activity, which drives CRC proliferation (109). In contrast, GAS5 interacts with YAP’s WW domain, promoting its cytoplasmic sequestration and inactivation, thereby suppressing CRC progression (110). Notably, circPPP1R12A encodes a functional peptide (circPPP1R12A-73AA) through an open reading frame (ORF), which directly activates YAP and significantly enhances CRC cell proliferation and migration (111). These ncRNAs modulate YAP’s localization, phosphorylation, ubiquitination, and transcriptional activity, significantly impacting CRC progression (**Table 2**).

**Table 2:** ncRNAs Targeting the Hippo Pathway to Improve CRC.

Non-Coding RNA		Interacting Molecule	Mechanism	Reference
LncRNAs	CAF-sEVsWEE2-AS1	MOB1A	Promotes the degradation of MOB1A, leading to YAP accumulation in the nucleus, activation of downstream gene transcription, CRC proliferation, and apoptosis inhibition.	(109)
	AGAP2-AS1	LATS1	Inhibits LATS1 phosphorylation, promotes YAP nuclear localization, and enhances transcriptional activity, driving CRC cell proliferation, migration, and invasion.	(108)
	GAS5	YAP	Directly interacts with the WW domain of YAP, promoting its cytoplasmic translocation, phosphorylation, and ubiquitin-mediated degradation, thereby suppressing CRC progression	(110)
	USP2-AS1	YAP	Reduces YAP phosphorylation levels and triggers the expression of downstream target genes in CRC cells, promoting proliferation and lung metastasis.	(112)
	B4GALT1-AS1	YAP	Facilitates YAP nucleo-cytoplasmic shuttling and transcriptional activity, enhancing CRC stem cell properties, migration, invasion, and the epithelial-mesenchymal transition (EMT) process.	(113)
miRNAs	miR-375-3p	YAP	Binds to specific sequences in the 3'-UTR of YAP mRNA, promoting its degradation and inhibiting protein synthesis, thereby suppressing CRC proliferation and increasing chemotherapy sensitivity.	(64)
	miR-429	LATS2	Downregulation of LATS2 kinase activity promotes YAP/TAZ expression and CRC progression.	(103)
	miR-103a-3p	LATS2, SAV1	Directly targets LATS2 and SAV1, enhancing TEAD1-YAP co-regulated HIF1A expression, promoting CRC cell proliferation, invasion, migration, glycolysis, and angiogenesis.	(56)
	miR-874-3p	YAP/TAZ	Downregulation directly targets YAP/TAZ, causing Hippo pathway inactivation and contributing to 5-FU resistance in CRC cells.	(107)
	miR-195	YAP	Targets the 3'-UTR of YAP mRNA to reduce YAP protein levels, inhibiting CRC cell proliferation, colony formation, invasion, and migration.	(104)
	miR-22-3p	KDM3A	Reduces YAP1 activity by targeting the 3'-UTR of KDM3A, suppressing CRC progression.	(106)
	miR-372-3p	LATS2	Binds to the 3'-UTR of LATS2, leading to mRNA degradation or translation inhibition, causing YAP nuclear accumulation and activation, promoting CRC proliferation, invasion, and downstream gene expression.	(105)
	miR-590-5p	YAP	Targets the 3'-UTR of YAP, suppressing its expression and thereby inhibiting CRC cell proliferation and invasion.	(114)
circRNAs	circPPP1R12A	YAP	Contains an open reading frame (ORF) capable of translating circPPP1R12A-73aa, which activates YAP, promoting CRC proliferation and metastasis.	(111)

Collectively, these epigenetic regulators—including DNA methylation, histone modifications, and ncRNAs—modulate key aspects of YAP/TAZ function, such as phosphorylate on, ubiquitination, subcellular localization, and transcriptional activity, thereby influencing CRC initiation, progression, and therapeutic response. While pharmacological modulation of these pathways remains at an early stage, accumulating evidence suggests that epigenetic targets, particularly ncRNAs, hold significant promise for future CRC therapy.

## Exploration of drugs targeting the Hippo/YAP pathway in CRC

Given the pivotal role of the Hippo/YAP signaling pathway in the development of CRC, targeting this pathway, specifically through inhibition of YAP expression and subcellular localization, has emerged as a promising therapeutic strategy. Various agents, including clinical drugs, molecular inhibitors, non-coding RNAs (ncRNAs), and natural products, have shown potential in modulating the Hippo pathway to suppress CRC progression.

### Clinical drugs

Several clinically used drugs have demonstrated significant anti-CRC effects by targeting YAP, Metformin, a widely used drug for type 2 diabetes, inhibits YAP nuclear function by promoting its phosphorylation, thereby reducing CRC cells’ immune evasion (115). Vilazodone, an antidepressant, blocks YAP nuclear localization and transcriptional activity, effectively decreasing CRC invasivenes (82). Simvastatin, a cholesterol-lowering agent, in combination with the EGFR inhibitor cetuximab, significantly suppresses tumor growth and extends survival, suggesting that inhibiting YAP activity is a key mechanism enhancing CRC treatment outcome (53).

### Clinical trial drugs targeting the Hippo/YAP pathway

In recent years, several novel agents specifically designed to target the Hippo-YAP/TAZ signaling axis have progressed into early-phase clinical trials, highlighting the therapeutic potential of this pathway in oncology.

For example, VT3989 is a first-in-class small molecule that binds to the palmitoylation pocket of TEAD transcription factors, thereby disrupting YAP/TAZ-TEAD interactions (116). In a first-in-human Phase I trial (NCT04665206) for advanced solid tumors enriched with NF2-mutant mesothelioma patients, VT3989 has demonstrated a tolerable safety profile and preliminary antitumor activity. Another TEAD inhibitor, IK-930 (117), is currently in a Phase I study (NCT05228015) in patients with advanced solid malignancies, after showing antitumor efficacy in preclinical models featuring Hippo pathway alterations. In addition, IAG933, a selective small-molecule inhibitor disrupting YAP/TAZ-TEAD protein-protein interactions, is being evaluated in a Phase I trial (NCT04857372) involving malignant mesothelioma and other tumors with NF2/LATS1/2 mutations or YAP/TAZ fusions (118). An antisense oligonucleotide targeting YAP1 (ION537), is also under investigation in a Phase I trial for advanced solid tumors (NCT04659096) (119). **Table 3** summarizes current therapies in clinical development that specifically target the Hippo/YAP pathway.

**Table 3:** Ongoing clinical trials targeting the Hippo/YAP pathway in cancer.

Drug	Target	Mechanism of Action	Trial Phase (Indication)	NCT Number	Reference
VT3989	TEAD inhibitor	Binds TEAD palmitoylation pocket to block YAP/TAZ–TEAD interaction	Phase I (Advanced solid tumors, NF2-mutant enriched)	NCT05228015	(116)
IK-930	TEAD inhibitor	Oral small-molecule inhibitor of TEAD palmitoylation; suppresses YAP/TAZ transcriptional output	Phase I (Advanced solid tumors)	NCT05228015	(117)
IAG933	YAP/ TEAD inhibitor	Selective disruptor of YAP–TEAD protein-protein binding	Phase I (Mesothelioma; NF2/LATS-mutant or YAP/TAZ fusion tumors)	NCT04857372	(118)
ION537	ASO to YAP1	Antisense oligonucleotide targeting YAP1 mRNA	Phase I (Advanced solid tumors)	NCT04659096	(119)

### Molecular inhibitors

Molecular inhibitors targeting YAP or its associated pathways have shown efficacy in reversing drug resistance and suppressing tumor growth in CRC. Verteporfin (VP), As a YAP inhibitor, VP reverses paclitaxel resistance in HCT-8/T cells and significantly enhances the sensitivity of LOVO/TAX cells to paclitaxel when combined with the drug (120, 121), VP also suppresses migration and invasion of DLD-1 cells (122) and further inhibits CRC progression when combined with the EGFR inhibitor AG1478, reversing chemotherapy resistance (51). Alisertib, an AURKA inhibitor, reduces YAP phosphorylation, restores cetuximab sensitivity, and suppresses CRC stemness (123). NUAK2 inhibitors, KHKI-01128 and KHKI-01215, suppress YAP transcriptional activity, thereby reducing CRC cell proliferation and inducing apoptosis in SW480 cells (124). Cyclovirobuxine D (CVB-D) enhances YAP phosphorylation, inhibits its nuclear translocation, and effectively suppresses CRC cell proliferation (125). These molecular inhibitors precisely modulate YAP activity and enhance the efficacy of anti-cancer drugs, providing a new perspective on overcoming CRC resistance.

### Natural products

Natural products and traditional Chinese medicine (TCM) extracts have garnered attention for their unique mechanisms in CRC treatment. These compounds modulate the Hippo/YAP signaling pathway through a variety of mechanisms, including targeted YAP, autophagy regulation, mitochondrial dynamics, pathway inhibition, and metabolic reprogramming, demonstrating their potential to inhibit CRC cell proliferation and invasion (**Table 4**).

**Table 4:** Natural Products Targeting YAP/Hippo Pathway in CRC Models.

Natural Product	Plant Source	Model	Dose	Expression of Related Factors	Reference
Calotropin	Calotropis gigantea	HT-29, HCT116 cells; BALB/c immunodeficient nude mice	0-10 µM;50 mg/kg	LATS1 ↓; YAP ↑; CTGF ↑	(126)
Ovatodiolide	Anisomeles indica	THP-1, HCT116, DLD-1 cells; NOD/SCID and BALB/c mouse models	*In vivo*: 5 mg/kg; with 5-FU: Ovatodiolide 5 mg/kg, 5-FU 30 mg/kg	YAP ↓; Kras ↓; mTOR ↓; IL-4 ↓; IL-6 ↓; ARG1 ↓; CD23 ↓	(127)
Resveratrol	/	HCT116 cells; athymic nude mice	*In vitro*: 20-80 µM; *In vivo*: 0, 50, 150 mg/kg	YAP ↓; pYAP ↑; CTGF ↓; CYR61 ↓; Bcl-2 ↓; Bad ↑; PCNA ↑	(128)
Curcumin	Curcuma longa	HCT116 and SW620 cells	2~16 µM	YAP ↓; LC3-II ↑; P62 ↓	(129)
Lappaol F	*Arctium lappa* seeds	HeLa, SW480, MDA-MB-231, PC3 cells; xenograft colon tumor mouse model	*In vitro*: 0-200 µg/mL; *In vivo*: 50 mg/kg, 100 mg/kg	14-3-3 ↑; YAP ↓; BIRC5 ↓; c-Myc ↓; Bcl-2 ↓	(130)
Procyanidins (OPC)	Grape seeds	HT29, HCT116 cells; immunodeficient mice	*In vitro*: 0-200 µg/mL; *In vivo*: 50 mg/kg, 100 mg/kg	LGR5 ↓; CD44 ↓; CD133 ↓; ZEB1 ↑; YAP ↓; TAZ ↓	(131)
Platycodin D	Platycodon grandiflorus	LoVo, OXA-R LoVo cells	1~20 μM	p-LATS2 ↓; p-YAP ↓; p21 ↑; p27 ↑	(132)
Toxicarioside G	Calotropis gigantea	SW480 cells	0.2 µM, 0.4 µM	LC3-II ↑; Beclin 1 ↑; Atg5 ↑; YAP ↑; LATS1 ↓	(133)
Physalin F	Physalis angulata	SW480, DLD1 cells; xenograft tumor nude mouse model	*In vitro*: 1-4 µM; *In vivo*: 10 mg/kg, 20 mg/kg	YAP ↑; Cyclin D1 ↓; c-Myc ↓; LEF1 ↓	(134)
Curcumol	Curcuma	HCT116, SW480, SW620, HCoEpiC cells; xenograft tumor mouse model	*In vitro*: 10-160 µg/mL; *In vivo*: 40 µg/mL	miR-30a-5p ↑; YAP1 ↓; β-catenin ↓; MMP2 ↓; E-cadherin ↑; MST-1 ↑; LATS1 ↑; p-YAP1 ↓	(135)
Magnolol	Magnolia	HCT116, SW480, DLD1, LS174T, RKO, HT29 cells; ICR mice	*In vitro*: 25 µM; *In vivo*: 5 mg/kg	DCLK1 ↓; LGR5 ↓; CD44 ↓; YAP1 ↑; TEAD ↓; PUMA ↑; Bcl2 ↓; BclXL ↓; Bax ↑; JNK ↑; ERK1/2 ↑	(136)
Matrine	Sophora flavescens	SW480 cells	1 nM; 5 nM	MIEF1 ↑; LATS2 ↑; Bax ↑; caspase-9 ↑; Bcl-2 ↓; survivin ↓; SOD ↓	(137)
Ginsenoside K	Panax ginseng	SW480, HT29, HCT116 cells; LoVo BALB/c nude mice	*In vitro*: 1-100 µM; *In vivo*: 50, 100, 200 mg/kg	PLA2G16 ↓; E-cadherin ↑; N-cadherin ↓; ZEB1 ↓; MMP-9 ↓; MMP-2 ↓; Vimentin ↓; p-YAP ↑; p-TAZ ↑	(18)
Tanshinone IIA	Salvia miltiorrhiza	SW480, SW837 cells	5 μM	Mst1 ↑; Bcl-2 ↓; Survivin ↓; Bax ↑; caspase-9 ↑; CDK4 ↓; Cyclin D1 ↓; Drp1 ↓; Fis1 ↓; Mfn2 ↓; OPA1 ↓	(113)
Ursolic Acid	/	HCT116, HT-29 cells; nude mice	*In vitro*: 5-20 µM; *In vivo*: 10 mg/kg/day	p-Akt ↓; p-Gsk3β ↓; C-Myc ↓; Rassf1A ↑; Mst1/2 ↑; Sav1 ↑; Mob1 ↑; p-Yap ↑; CTGF ↓	(138)

YAP plays a central role in tumor cell proliferation and invasion, Calotropin promotes LATS1 degradation, leading to YAP dephosphorylation and nuclear localization (126). Similarly, Ovatodiolide inhibits the expression of YAP-regulated M2 tumor-associated macrophage polarization factors, such as IL-4 and IL-13, altering the tumor microenvironment and thereby regulating the progression of CRC (127). Resveratrol increases YAP phosphorylation levels while decreasing YAP protein, disrupting YAP-TEAD interactions and inhibiting HCT116 cell proliferation (128). Lappaol F regulates the post-translational modification of YAP through its interaction with 14-3-3 (130). Oligomeric proanthocyanidins downregulate YAP/TAZ, thereby inhibiting the expression of colorectal cancer stem cell markers LGR5, CD44, and CD133 (131). Platycodin D activates the LATS2/YAP1 axis, inhibiting nuclear transcriptional activation of YAP1 and enhancing oxaliplatin sensitivity (132). Curcumol upregulates miR-30a-5p, promoting YAP and TAZ phosphorylation and inactivation (135). Honokiol interacts with PUMA, leading to the sequestration of YAP in the cytoplasm, thereby further suppressing its oncogenic activity (136).

Curcumin enhances autophagy by reducing YAP protein levels, upgrading autophagy marker LC3-II, and promoting P62 degradation, augmenting its anti-CRC effects (129). Toxicarioside G (TCG) blocks autophagic flux, inducing autophagosome accumulation and YAP activation. This bidirectional regulatory strategy positions TCG as a potential CRC inhibitor (133).

Matrine and Tanshinone IIA activate the upstream Hippo kinases LATS2 and LATS1, respectively. These promote MIEF1-mediated and INF2-mediated mitochondrial fission, effectively suppressing CRC cell survival and inducing apoptosis (113, 139).

Inhibition of signaling pathways is another important therapeutic strategy. Physalin F accelerates β-catenin ubiquitination and degradation by promoting YAP interaction with the β-catenin degradation complex, inhibiting the Wnt/β-catenin signaling pathway in a YAP-dependent manner (134). Ursolic acid combined with Doxorubicin inhibits the YAP/TAZ signaling pathway by promoting YAP degradation while simultaneously blocking the Akt/GSK3β signaling pathway, demonstrating the potential of multi-target interventions in cancer treatment (138).

Finally, Ginsenoside K exerts its anti-CRC effect by inhibiting PLA2G16 protein, which may regulate metabolic reprogramming of tumor cells through Hippo signaling pathway (18).

These studies demonstrate that naturally occurring active compounds can have anticancer effects in CRC by modulating Hippo signaling pathways that regulate proliferation, apoptosis, autophagy, stemness, and tumor microenvironment. Additionally, TCM formulations and crude extracts have also shown potential in CRC inhibition.

The Jianpi Huatan Formula (JPHTF), a traditional TCM prescription, has been proven effective as an adjuvant therapy for CRC, particularly in improving postoperative quality of life in stage II/III patients. In patient-derived xenograft (PDX) models of CRC with RAS mutations, JPHTF improved CRC treatment outcomes by modulating both the Hippo and Hedgehog signaling pathways (140).

Extracts from Polygonum barbatum (PBE) and Ilex rotunda Thunb (WIR) have demonstrated potential in CRC treatment. PBE inhibits YAP1 phosphorylation at Ser127, preventing its interaction with TEAD transcription factors in the nucleus, thereby suppressing YAP-mediated oncogene expression. PBE also affects Wnt signaling by interacting with β-catenin, weakening CRC stem cell properties through a synergistic effect with the Hippo/YAP pathway (141). WIR reduces inflammatory cytokines IL-6 and TNF-α, indirectly regulating miR-31-5p expression. This restores LATS2 expression in the colonic tissue and inhibits YAP accumulation. Through anti-inflammatory effects and modulation of the miR-31-5p/YAP pathway, WIR suppresses CRC stem cell properties, highlighting its potential as a preventive herbal product against CRC (142).

Natural active compounds, TCM formulations, and crude extracts offer diverse strategies for CRC treatment by targeting multiple biological mechanisms. In conclusion, these natural active compounds, traditional Chinese medicine formulas, and crude extracts modulate multiple biological mechanisms via the Hippo/YAP signaling pathway, providing a diverse array of options for the development of therapeutic agents against CRC.

## Discussion and conclusion

The Hippo signaling pathway is a highly conserved regulatory cascade across diverse species and plays a pivotal role in CRC by modulating cellular proliferation, invasion, metastasis, and shaping the tumor microenvironment. Importantly, it also influences the sensitivity of tumors to targeted therapies. Elevated expression of YAP has been widely implicated in CRC initiation and progression. Recent advances in clinical trial data, epigenetic regulation, and insights into the dual functions of YAP/TAZ have significantly deepened our understanding of the Hippo/YAP axis in CRC pathophysiology.

Currently, several YAP-targeted inhibitors are undergoing Phase I clinical evaluation and have shown promising translational potential. At the same time, the emerging evidence of the context-dependent tumor-suppressive roles of YAP highlights the need to exercise caution when designing therapeutic strategies. While YAP and TAZ are typically regarded as oncogenic drivers, under specific conditions, they may also exert tumor-suppressive effects. In β-catenin-driven CRC models, for example, YAP activation has been shown to inhibit Wnt signaling and promote tumor cell differentiation. Mechanistically, nuclear YAP/TAZ can stably associate with Groucho/TLE transcriptional co-repressors at Wnt target gene promoters, thereby attenuating β-catenin–TCF4 transcriptional activity. This interaction leads to the loss of stem-like features, enhanced differentiation, and suppression of aberrant Wnt-driven proliferation (143). Similarly, in APC-mutant CRC models, enforced YAP activation reprograms cancer stem cells into a low-Wnt, non-proliferative, differentiated phenotype, resulting in regression of both primary and metastatic lesions (144). Conversely, YAP deletion has been shown to accelerate Wnt-mediated tumorigenesis in the intestinal epithelium. These findings underscore the dualistic and context-specific roles of YAP, which must be carefully considered when developing YAP-targeted therapies.

As our mechanistic understanding of the Hippo pathway continues to evolve, research focusing on upstream kinases and pharmacological YAP inhibitors has broadened the therapeutic landscape of CRC. These efforts are expected to uncover novel regulatory mechanisms and accelerate the development of more effective therapeutic strategies. Ultimately, the integration of Hippo/YAP signaling insights into clinical practice holds the potential to overcome current treatment bottlenecks and achieve the goals of personalized and precision medicine in CRC.
